# Predicting Deviant Behaviors in Sports Using the Extended Theory of Planned Behavior

**DOI:** 10.3389/fpsyg.2021.678948

**Published:** 2021-09-09

**Authors:** Sangwook Kang, Inwoo Kim, Keunchul Lee

**Affiliations:** ^1^Department of Physical Education, Seoul National University, Seoul, South Korea; ^2^Department of Sports Culture, Dongguk University Seoul, Seoul, South Korea; ^3^Department of Physical Education, Changwon National University, Changwon, South Korea

**Keywords:** athlete, deviant behavior, theory of planed behavior, impulsivity, moral obligation

## Abstract

The purpose of the present study is to examine the deviant behaviors of young athletes the using extended theory of planned behavior (TPB) and impulsivity. About 536 middle and high school athletes in South Korea answered a set of questionnaires that measured their attitude, subjective norms, perceived behavioral control, intention, ethical obligation, and impulsivity. Structural equation model (SEM) analysis revealed that the extended TPB model is adequate to explain deviant behaviors in sports. Further, the underlying intentions that motivate the deviant behaviors of athletes in sports were significantly predicted by perceived behavioral control and moral obligation. Findings also suggested that the intention for deviant behaviors in sports more readily manifests as an actual act when the impulsivity scores are high. Theoretical contributions and practical implications are addressed in the Discussion section.

## Introduction

Participation in sports can promote ethical behavior and the development of healthy morals (Weiss and Bredemeier, 1990; Shields and Bredemeier, 1995; Clifford and Feezell, 1997). However, unfair play and other unethical behaviors that would otherwise cause problems in everyday life are often overlooked in sports settings because the athletes tend to justify such behaviors by believing that the result says everything or that they must win by fair or foul means in competitions (Mallia et al., 2019). With regard to this issue, Beller and Stoll (1995) reported that non-athletes more frequently applied greater reasoning in their approaches to moral dilemmas than athletes. A similar research indicated that high school and college athletes exhibited lower moral attitudes than non-athlete students (Shields and Bredemeier, 1995).

Low moral attitudes among athletes can lead to deviant behaviors in sports settings, such as intentionally injuring opponents (Kavussanu et al., 2006), cheating the referees (Shields et al., 2005), blaming teammates (Kavussanu and Boardley, 2009), and faking an injury to get ahead in the game (Long et al., 2006). Such unethical behaviors have been historically known to be disregarded in sports contexts, where the disproportionate emphasis is placed on competitiveness compared to fairness (Sherif, 1978), and many athletes do not consider these immoral behaviors as significant, relative to their athletic achievement (Smith, 1979).

Today, social awareness toward unethical behaviors in sports has changed, as have the personal responses of people to these deviant behaviors. People consider malicious violations or fouls as morally reprehensible, even if those behaviors contributed to athletic victory. For example, Thierry Henry, a French soccer hero, was harshly criticized by his own countrymen for his ignominious handball foul for France against the Republic of Ireland in the World Cup play-off in 2009. Although this behavior led to France's qualification for the 2010 FIFA World Cup, the results of a public survey in France showed an 88% disapproval rate (France Télévisions., 2009). Also, the French teachers' union issued a statement condemning the unethical behavior of Henry and criticized the attitude that winning is the most important thing in sports. Sometimes, it is the general public and the fans that implicitly encourage the athletes to engage in immoral behavior.

Although public awareness toward moral behaviors in sports has shifted such that a sense of fair play is considered more important than winning, such deviant behaviors are still prevalent in sports competitions (Mallia et al., 2019). Athletes and coaches do not adhere to the rules of the field or court. In fact, a study indicated that exclamations from the coaches during a Judo match influence the decisions of referees so as to favor their own athletes (Souchon et al., 2013). There are some coaches who encourage the players to intimidate or even injure their opponents at the beginning of the match. Similarly, some baseball coaches instruct their catchers to chat with the opponent batters so as to distract them when they are waiting for the ball. Since the players willingly engage in these behaviors, it is evident that deviant behaviors in sports are partly accepted by both the coaches and the players (Conroy et al., 2001; Park et al., 2019).

Many studies have been conducted to understand such deviant behaviors. According to Lance (2005), athletes tend to learn and use deviant behaviors depending on the social norms and values that exist in their sports. Smith (1979) also suggested that violent behaviors typically emerge from the values and attitudes of athletes toward deviant behaviors in sports. One study that examined the predictors of poor sports behaviors revealed that moral awareness, such as a sense of justice, influences the actual behaviors, and such poor behaviors tend to be more pronounced when the athletes perceive the deviant behaviors of their peers and coaches (Shields et al., 2007). Hence, it is evident that the intentions of deviant behaviors in sports are influenced by different factors, such as an individual's attitude, moral awareness, and perception of the behaviors of others.

Various theories have been used to explain deviant behaviors in sports. According to a review study (Kavussanu, 2008) that investigated moral behaviors in sports, common models such as the strain theory (Merton, 1968), the four-component model (Rest, 1986), and the social cognitive theory (Bandura, 1991) have been used by researchers to interpret these behaviors. Although each theory explains the behaviors of athletes in a different way, there is a consensus as to which internal process of the human mind should be crucially considered to understand their behaviors.

The strain theory, first proposed by Merton (1968), suggests that when individuals experience a conflict between goals and means, some opt to achieve the goals through illegal means, while others who are more appropriately socialized alleviate this stress by adjusting their aspirations so that the goals are more practically attainable. However, this view is limited in its ability to explain deviant behaviors in the context of sports because a breach of conduct by athletes is not always due to a conflict between goals and means (Lee and Lim, 1999). Lee and Lim criticized Merton (1968) strain theory on the grounds that the theory regards deviant behaviors as a reaction, as opposed to independent actions backed by free will.

The theory of planned behavior (TPB) (Ajzen, 1991), which is built around perceived behavioral control, was designed to overcome the limitations of reasoned action theory. Perceived behavioral control is defined as an individual's perception of the ease or difficulty in demonstrating a particular behavior. The concept was developed to address the idea that behavioral intention alone is not sufficient to trigger a subsequent action, due to other situational factors. Perceived behavioral control plays a role similar to that of self-efficacy in social cognitive theory (Bandura, 1991). For a behavioral intention to materialize into an act, available opportunities and resources (e.g., time, money, skills, and cooperation from others) must support an individual's motivation. Furthermore, opportunities and resources influence perceived behavioral control (Ajzen, 1991). In short, behavioral intention increases when an individual's attitude and subjective norms favor an act, and perceived behavioral control is high. Research has demonstrated a strong association among attitude, subjective norms, perceived behavioral control, and behavior (Schifter and Ajzen, 1985; Ajzen and Madden, 1986).

The theory of planned behavior has been considered a useful theoretical framework to predict human behaviors (Ajzen and Fishbein, 2005; Conner and Sparks, 2005). However, many researchers have criticized the theory for its insufficient or inadequate explanation of human social behaviors; further work is thus necessary to complement or improve upon the theory. The results of a meta-analysis concerning TPB indicated that an extended model of TPB that includes other variables, such as personalities of individuals, should be implemented to increase the predictive power of the model (Armitage and Conner, 2001; Ajzen, 2011). In a 2008 review, Kavussanu also advised that future research should explore different latent variables to predict moral behaviors in sports.

The present study applied the extended TPB to broaden our understanding of deviant behaviors in sports. First, an individual's sense of moral obligation to build on the basic model of the TPB was considered in an effort to understand the moral behaviors of athletes (Schwartz and Tessler, 1972; Gorsuch and Ortberg, 1983; Beck and Ajzen, 1991). Based on the results of existing studies in which the moral disposition of athletes was shown to influence immoral behaviors (Kavussanu et al., 2002; Kavussanu and Spray, 2006), it was concluded that a sense of moral obligation can predict unethical behaviors, such as deviant behaviors in sports.

Secondly, we considered the possibility that impulsivity is related to the deviant behaviors of athletes. Subjective expected utility theory has been criticized as a basis of TPB since it excludes the effects of the affective and impulsive factors of individuals on the decision-making process. Impulsivity refers to the inability or unwillingness to think about the consequences of behavior before deciding to act (Buss and Plomin, 1975). The greater the behavioral intention, the greater the chance of enactment. However, the TPB loses its predictive utility when it comes to impulsive acts (Beck and Ajzen, 1991). Indeed, people sometimes act without concern for the negative consequences of their behaviors (Avila, 2001), and another relevant study indicated that impulsivity moderates the impact of intention on the actual behaviors of individuals (Churchill and Jessop, 2010). Thus, impulsivity appears to affect the pathways from the underlying intentions of athletes' deviant behaviors to their actual behaviors in sport.

Hence, the first aim of the present study was to examine whether the inclusion of moral behavior in the extended TPB could predict the deviant behaviors of young athletes in sports. Such a model could suggest further appropriate theoretical frameworks within which to understand the immoral behaviors of young athletes in the related sports settings. The second aim of this study was to investigate the moderating effect of impulsivity on the relationship between the intentions of the athletes and their actual behaviors. The study will provide an insight on how to handle athletes' deviant behaviors during competitions.

Hypothesis 1. The addition of moral obligation to the extended TPB will positively predict the deviant behaviors of young athletes in sports.

Hypothesis 2. Impulsivity will moderate the relationship between intention and behaviors.

## Materials and Methods

### Participants

To prevent the bias toward a particular sport and a specific gender, the ratio of sport type to gender registered with the Korea Sports Association was calculated, and a stratified sample method was conducted until all the participants matching the ratio were recruited. A total of 554 (men, *n* = 363; women, *n* = 191) athletes have participated at Time 1, out of which 18 of them dropped out and 536 (men, *n* = 350; women, *n* = 186) have participated in various sports (athletics, gymnastics, swimming, taekwondo, judo, boxing, wrestling, shooting, archery, badminton, weightlifting, cycling, fencing, curling, etc.) at Time 2 (*M* age = 16.49 years; *SD* = 1.50). All participants were selected from five middle and high schools for sports, registered with the Korean Sports Association, which has been driving forces in fostering the growth and careers of elite sports athletes. These middle and high schools for sports in Korea are educational institutions that nurture elite athletes to compete at the national level. However, the pressure to perform at a high level coupled with fierce competition are causing extreme stress on the athletes (Lee et al., 2017), resulting in side effects such as burnout and deviant behaviors (Park et al., 2019).

### Study Procedure

After obtaining permission from the heads of the institutions, the recruitment documents for research participants were posted on the bulletin boards of each school in Seoul, Gangwon, and Gwangju. Students volunteered to participate in the research after reading the research description, and they were allowed to participate only after submission of both the research participation consent form and the parental consent form. We provided a concise information session on the study in each school where the study participants were recruited. The participants were each assigned an identification (ID) for follow-up studies, after which the questionnaires were distributed. This study involved collection of data at two time points. We collected responses pertaining to impulsivity, attitude, subjective norms, perceived behavioral control, intention, and moral obligation at Time 1 (June 2020) were collected. We visited the school once again at Time 2 (October 2020) and collected information regarding deviant behaviors observed over 3 months. The completed questionnaires were collected and the data were analyzed. The research was conducted in accordance with the principles set forth in the Declaration of Helsinki, and all the procedures were approved by the Seoul National University of Institutional Review Board (IRB No. 2103/001-007).

### Measures

#### Impulsivity Questionnaire

The Barrat Impulsivity Scale-15 (Barratt, 1959; Spinella, 2007) was used to measure impulsivity. This scale consists of 15 questions, including five questions on motor impulsivity, five questions on non-planning, and five questions on attention impulsivity. Examples of questions for each factor included the following statements: “I act on the spur of the moment” for motor impulsivity, “I plan for the future [inverted]” for non-planning, and “I am restless at lectures or talks” for attention impulsivity. A 5-point Likert scale (1 = absolutely not, 5 = very much so) was used for every question. The confidence interval (Cronbach's alpha) ranged from 0.802 to 0.818. Maximum likelihood method (MLM) was used for the primary confirmatory factor analysis on the 15 questionnaire items. One item was eliminated because the standardized regression coefficient was lower than the threshold value (0.50). A secondary confirmatory factor analysis on the remaining 14 items and the covariance between the error terms among questions 6 and 7 was established (Jöreskog and Sörbom, 2012). The fitness indices, including χ^2^ = 210.696 (*df* = 73, *p* < 0.001), comparative fit index [CFI] = 0.958, Tucker–Lewis index [TLI] = 0.948, and root mean square error of approximation [RMSEA] = 0.059, were within the acceptable limits (Hu and Bentler, 1999). Based on these analyses, the final impulsivity questionnaire was on attention impulsivity.

#### Extended TPB Questionnaire

Items first developed by Beck and Ajzen in a study conducted in 1991 were modified in the present questionnaire to measure the attitude, subjective norms, perceived behavioral control, intention, and moral obligation. The questionnaire included three bipolar adjective items (namely, good–bad, foolish–wise, and useful–useless) for attitude, three items for subjective norms, four items for perceived behavioral control, three items for intention, three items for moral obligation, and four items for behavior. The behavior items addressed how often respondents engaged in behaviors such as rule-bending, unsportsmanlike behavior, verbal violence, and physical violence. To measure the deviant behaviors of the participants more accurately, we used modified items which were adapted by Anderman et al. (1998) from the original scale of Beck and Ajzen (1991).

The participants were asked to reflect on their deviant behaviors in sports over the course of the preceding year. The participants then answered questions designed to evaluate their behaviors. Examples of items for each variable are provided for each category. For example, an item from the “Attitude” category read: “My overall attitude toward deviant behaviors in sports is good (or bad)”. To assess “Subjective norms”, the statement: “My coach and teammates think that it is ok to engage in deviant behaviors during a game”, was presented. For “Perceived behavioral control”, the respondents were asked how strongly they agree with the statement: “I could pull off a deviant behavior during a game, if I wanted to”. For “Intention”, an example item was: “I would never conduct deviant behaviors in sports”. To ascertain “Moral obligation”, the participants were asked to respond to the statement: “Engaging in deviant behaviors in sports undermines my personal codes of ethics”. For “Behavior”, the statement: “Past 3 months, sometimes I broke the rules when I thought it was necessary”, was presented. Each item was coded on a 5-point Likert scale (1 = absolutely not, 5 = very much so). Cronbach's alphas were 0.843 for attitude, 0.833 for subjective norms, 0.800 for perceived behavioral control, 0.812 for intention, 0.815 for moral obligation, and 0.938 for behavior. MLM was used for the primary confirmatory factor analysis on the 20 items. One item was eliminated (Intention 2) because the standardized regression coefficient was lower than the threshold value of 0.50. A secondary confirmatory factor analysis was performed on the remaining 19 items, which indicated that all fit indices were acceptable: χ^2^ = 529.433 (*df* = 137, *p* < 0.001), CFI = 0.942, TLI = 0.928, and RMSEA = 0.073. The final items in the extended model of TPB included three items for attitude, three items for subjective norms, four items for perceived behavioral control, three items for moral obligations, two items for intention, and four items for behavior.

### Analysis

First, a confirmatory factor analysis was performed to verify the measurements' validity, and Cronbach's alpha was calculated as a measure of reliability. To examine the global characteristics of the data, a correlation analysis was performed and descriptive statistics were computed. SEM analysis was used to measure model fit indices. To examine the effects of impulsivity on deviant behaviors in sports using SEM, the respondents were divided into two groups (low and high) according to the median scores on the three factors related to impulsivity. Prior to comparing the groups' path coefficients, configural invariance and metric invariance were tested. A χ^2^ test was performed comparing the unrestricted model and a model where the effect of intention on deviant behavior was restricted to be the same across groups. The significance level was set at 0.05.

## Results

### Descriptive Statistics and Correlations Between Observed Variables

The results of the correlation analysis involving attitude, subjective norms, perceived behavioral control, moral obligation, intention, behavior, and impulsivity are shown in Table 1. Moral obligation was negatively correlated with the other variables, and all other variables were positively correlated with each other (Table 1).

**Table 1:** Correlation matrix and descriptive statistics (*N* = 536).

	**a1**	**a2**	**a3**	**s1**	**s2**	**s3**	**p1**	**p2**	**p3**	**p4**	**i1**	**i2**	**m1**	**m2**	**m3**	**b1**	**b2**	**b3**	**b4**	**M (SD)**
a1	1	0.624^**^	0.651^**^	0.313^**^	0.422^**^	0.401^**^	0.431^**^	0.303^**^	0.317^**^	0.235^**^	0.426^**^	0.394^**^	−0.384^**^	−0.369^**^	−0.403^**^	0.234^**^	0.229^**^	0.185^**^	0.183^**^	2.3 (1.1)
a2	0.622^**^	1	0.615^**^	0.326^**^	0.426^**^	0.396^**^	0.451^**^	0.233^**^	0.330^**^	0.278^**^	0.406^**^	0.336^**^	−0.351^**^	−0.283^**^	−0.294^**^	0.139^*^	0.152^*^	0.101	0.143^*^	2.3 (1.1)
a3	0.671^**^	0.624^**^	1	0.365^**^	0.644^**^	0.598^**^	0.569^**^	0.361^**^	0.378^**^	0.409^**^	0.543^**^	0.396^**^	−0.528^**^	−0.474^**^	−0.446^**^	0.266^**^	0.305^**^	0.233^**^	0.253^**^	2.0 (0.98)
s1	0.366^**^	0.309^**^	0.440^**^	1	0.609^**^	0.445^**^	0.391^**^	0.298^**^	0.267^**^	0.153^*^	0.311^**^	0.158^**^	−0.308^**^	−0.227^**^	−0.261^**^	0.070	0.084	0.079	0.062	1.9 (0.91)
s2	0.395^**^	0.333^**^	0.560^**^	0.727^**^	1	0.685^**^	0.558^**^	0.356^**^	0.339^**^	0.330^**^	0.478^**^	0.323^**^	−0.441^**^	−0.330^**^	−0.333^**^	0.207^**^	0.266^**^	0.182^**^	0.195^**^	1.7 (0.82)
s3	0.343^**^	0.313^**^	0.536^**^	0.538^**^	0.664^**^	1	0.470^**^	0.378^**^	0.383^**^	0.315^**^	0.421^**^	0.393^**^	−0.406^**^	−0.333^**^	−0.352^**^	0.285^**^	0.270^**^	0.219^**^	0.228^**^	1.8 (0.88)
p1	0.420^**^	0.373^**^	0.525^**^	0.388^**^	0.494^**^	0.508^**^	1	0.509^**^	0.485^**^	0.457^**^	0.523^**^	0.422^**^	−0.491^**^	−0.492^**^	−0.435^**^	0.212^**^	0.312^**^	0.211^**^	0.239^**^	1.9 (0.90)
p2	0.273^**^	0.170^**^	0.349^**^	0.286^**^	0.362^**^	0.459^**^	0.458^**^	1	0.616^**^	0.338^**^	0.471^**^	0.418^**^	−0.405^**^	−0.328^**^	−0.269^**^	0.283^**^	0.273^**^	0.179^**^	0.300^**^	2.1 (1.1)
p3	0.257^**^	0.170^**^	0.332^**^	0.271^**^	0.337^**^	0.384^**^	0.437^**^	0.681^**^	1	0.394^**^	0.527^**^	0.477^**^	−0.371^**^	−0.337^**^	−0.313^**^	0.250^**^	0.266^**^	0.186^**^	0.269^**^	2.2 (1.0)
p4	0.187^**^	0.145^*^	0.360^**^	0.165^**^	0.326^**^	0.299^**^	0.328^**^	0.490^**^	0.450^**^	1	0.588^**^	0.484^**^	−0.481^**^	−0.509^**^	−0.338^**^	0.238^**^	0.254^**^	0.157^**^	0.206^**^	2.0 (0.97)
i1	0.288^**^	0.201^**^	0.392^**^	0.378^**^	0.515^**^	0.488^**^	0.504^**^	0.513^**^	0.454^**^	0.487^**^	1	0.753^**^	−0.643^**^	−0.649^**^	−0.450^**^	0.331^**^	0.338^**^	0.184^**^	0.328^**^	1.8 (0.83)
i2	0.317^**^	0.198^**^	0.407^**^	0.296^**^	0.344^**^	0.367^**^	0.474^**^	0.425^**^	0.426^**^	0.375^**^	0.564^**^	1	−0.546^**^	−0.508^**^	−0.399^**^	0.384^**^	0.331^**^	0.266^**^	0.332^**^	2.0 (0.95)
m1	−0.263^**^	−0.162^**^	−0.354^**^	−0.318^**^	−0.436^**^	−0.394^**^	−0.452^**^	−0.430^**^	−0.387^**^	−0.462^**^	−0.626^**^	−0.499^**^	1	0.696^**^	0.591^**^	−0.308^**^	−0.268^**^	−0.171^**^	−0.252^**^	4.1 (0.84)
m2	−0.246^**^	−0.115	−0.320^**^	−0.238^**^	−0.387^**^	−0.343^**^	−0.436^**^	−0.386^**^	−0.355^**^	−0.453^**^	−0.556^**^	−0.431^**^	0.624^**^	1	0.588^**^	−0.200^**^	−0.228^**^	−0.115	−0.217^**^	4.0 (0.90)
m3	−0.257^**^	−0.099	−0.247^**^	−0.193^**^	−0.288^**^	−0.218^**^	−0.296^**^	−0.264^**^	−0.244^**^	−0.258^**^	−0.328^**^	−0.268^**^	0.412^**^	0.551^**^	1	−0.276^**^	−0.261^**^	−0.234^**^	−0.208^**^	3.7 (1.0)
b1	0.181^**^	0.158^*^	0.331^**^	0.319^**^	0.429^**^	0.436^**^	0.441^**^	0.389^**^	0.413^**^	0.336^**^	0.518^**^	0.333^**^	−0.459^**^	−0.384^**^	−0.220^**^	1	0.818^**^	0.650^**^	0.760^**^	1.3 (0.63)
b2	0.206^**^	0.183^**^	0.393^**^	0.369^**^	0.492^**^	0.479^**^	0.419^**^	0.377^**^	0.412^**^	0.331^**^	0.517^**^	0.358^**^	−0.443^**^	−0.378^**^	−0.252^**^	0.889^**^	1	0.703^**^	0.808^**^	1.3 (0.56)
b3	0.206^**^	0.183^**^	0.283^**^	0.329^**^	0.388^**^	0.305^**^	0.403^**^	0.314^**^	0.329^**^	0.206^**^	0.356^**^	0.277^**^	−0.374^**^	−0.301^**^	−0.241^**^	0.698^**^	0.721^**^	1	0.739^**^	1.2 (0.53)
b4	0.228^**^	0.228^**^	0.391^**^	0.391^**^	0.456^**^	0.426^**^	0.487^**^	0.307^**^	0.272^**^	0.278^**^	0.415^**^	0.313^**^	−0.422^**^	−0.308^**^	−0.245^**^	0.716^**^	0.781^**^	0.780^**^	1	1.2 (0.47)
M (SD)	2.7 (1.0)	2.6 (1.0)	2.4 (0.99)	2.3 (0.94)	2.1 (0.90)	2.2 (0.92)	2.3 (0.84)	2.7 (1.0)	2.7 (0.92)	2.4 (0.94)	2.3 (0.92)	2.5 (0.89)	3.6 (0.85)	3.5 (0.85)	3.3 (0.89)	2.0 (0.97)	1.9 (0.92)	1.9 (0.96)	1.8 (0.89)	

*All variables are students' ratings of perceptions*.

*a, attitude; s, subjective norm; p, perceived behavioral control; I, intention; m, moral obligation; b, behavior; M (SD), mean (standard deviation); Lower, high impulsivity group (259); Upper, low impulsivity group (277)*.

* *p < 0.05*,

** *p < 0.01*.

### Testing the Adequacy of the Extended Model of TPB

**Figure 1:**
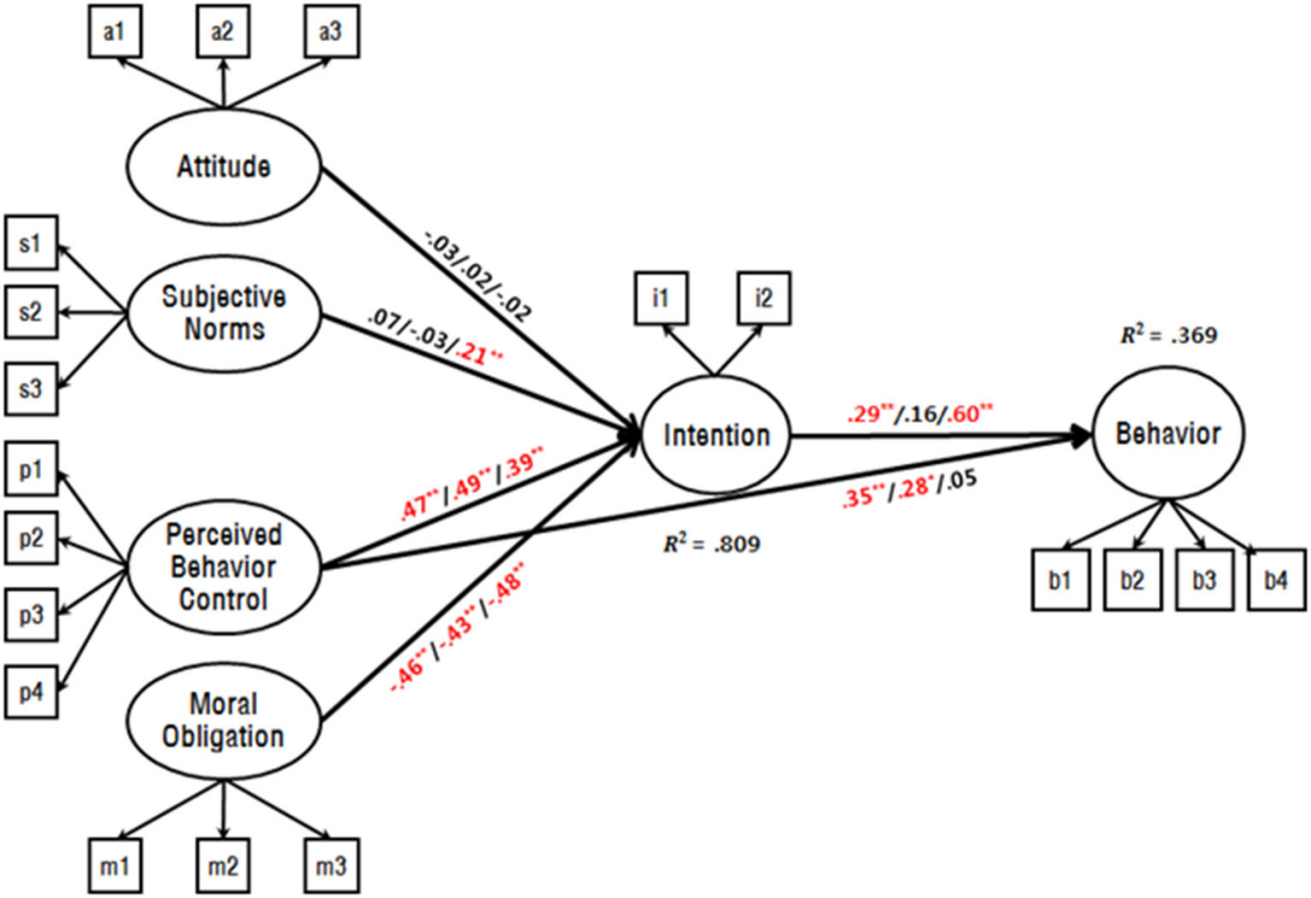
Path coefficient of extended theory of planned behavior (TPB) model with impulsivity level (Non-grouping/Low/High). Values above/below arrows represent standardized path coefficients. Values above/below latent variable represent SMC value. Covariance pathways were estimated among attitude, subjective norms, perceived behavior control, and moral obligation, but excluded from the figure for clarity. ^*^p < 0.05, ^**^p < 0.01.

Structural equation modeling (SEM) analysis was used to test the adequacy of the extended model of TPB that had moral obligation as an additional variable to predict unethical behaviors with greater accuracy (Schwartz and Tessler, 1972; Gorsuch and Ortberg, 1983; Beck and Ajzen, 1991). Maximum likelihood method (MLM) was used for factor estimation. Results of a goodness-of-fit test for the extended model of TPB (χ^2^ = 541.541, *df* = 140, *p* < 0.001, CFI = 0.941, TLI = 0.928, and RMSEA = 0.067–0.080) were within the acceptable ranges of values. Four predictive variables, including moral obligation (*R*^2^ = 0.809), accounted for the intention of behaviors (~81%) and explained the variance of athlete behavior, which was calculated as *R*^2^ = 0.369. These results lead us to conclude that the extended model of TPB is appropriate to predict deviant behaviors in sports. The standardized path coefficients are shown in Figure 1. All pathways in the model, prior to the final model verification, were significant. Figure 1 is a schematic representation of the research model, and the fit of this model was satisfactory.

### Effects of Intention on Deviant Behaviors in Sports Based on Impulsivity Level

To compare the path coefficients (moderate effectiveness) for the effect of intention on deviant behaviors according to impulsivity level, the participants were divided into groups. The median score for the three factors pertaining to impulsivity was 2.53, and the standard deviation was 0.535. Participants who scored below the average (*n* = 277) were classified as “low impulsivity”, and those who scored above the average (*n* = 259) were classified as “high impulsivity”. SEM analysis was used for a multi-group analysis of the extended model of TPB. MLM was used for the factor estimation.

Since the research model did not consider the mean differences of some factors between the groups divided by impulsivity, there is a possibility of correlation error (Nunnally et al., 1967). Therefore, prior to comparing the path coefficients between groups, an invariance test was performed to ensure that the measurement scale was being applied equally in both groups. Table 2 shows the fit indices for the configural invariance model (no restriction between groups) and the metric invariance model (effects of latent variables restricted to be identical across groups). For configural invariance, χ^2^ = 1247.64 (*df* = 420, *p* < 0.001); therefore, null hypothesis was rejected. The values of CFI (0.936), TLI (0.922), and the RMSEA confidence interval (0.040–0.046) were adequate, indicating that the conditions for configural invariance were satisfied. For metric invariance, χ^2^ = 1269.398 (*df* = 446, *p* < 0.001); therefore, the null hypothesis was rejected. The values of CFI (0.936), TLI (0.927), and the RMSEA confidence interval (0.039~0.044) were adequate. A χ^2^ test comparing models 3 and 4 yielded Δχ^2^ = 21.753 and Δdf = 26, indicating no rejection of the null hypothesis. Subsequently, metric invariance was verified. Because metric invariance was confirmed, we tested whether paths (β) that led from intention to deviant behaviors in sports were influenced by impulsivity level. A χ^2^ test comparing the β path restriction and configural invariance models revealed Δχ^2^ = 7.091 and Δdf = 1; therefore, the null hypothesis was rejected (*p* < 0.05). This indicates that the β path coefficient indices were statistically different between the low (0.16) and high (0.60) impulsivity groups. The standardized path coefficients are shown in Figure 1.

**Table 2:** Goodness of fit of the configural invariance model, metric invariance model, and constrained model (*N* = 536).

**Group**	**Model**	**χ^2^**	**df**	**Δχ^2^**	***p***	**CFI**	**TLI**	**RMSEA (C.I.)**
Impulsivity	Configural invariance	1247.64	420			0.936	0.922	0.040–0.046
	Metric invariance	1269.39	446	21.75	0.70	0.936	0.927	0.039–0.044
	β path Constrained	1254.73	421	7.09	0.00	0.936	0.922	0.040–0.046

*All variables are measure of perceptions. Δχ^2^ = chi-square difference*.

*Configural invariance = all paths were freely estimated. Metric invariance = all factor estimates are constrained to be identical between groups. β path constrained = the path from intention to behavior was constrained to be identical between groups*.

## Discussion

The present study assumed that an individual's moral disposition influences deviant behaviors in sports, and examined the validity of an extended model of the TPB which includes “moral obligation” as a predicting variable. In the extended model, the figures of squared multiple correlation (SMC) (0.809) and RMSEA confidence interval (0.067–0.080) were acceptable. The factors assessed here explained 80.9% of intention and 36.9% of behaviors. This indicates that the extended model of TPB that takes moral obligation into account is an adequate theoretical framework within which to understand and predict deviant behaviors in sports.

The results also suggested that perceived behavioral control and moral obligation were significant factors underlying the intention of deviant behaviors in sports, while attitude and subjective norms were not significant. According to an earlier study based on TPB (Ajzen, 2011), the relative weights of preceding factors imply the strength with which these factors affect intentions. Thus, we can infer that perceived behavioral control and moral obligation significantly influence the intention of such actions. Therefore, to reduce the athletes' intention of engaging in deviant behaviors in sports, interventions should be aimed at undermining the perception of behavioral control and promoting the sense of moral obligation related to such behaviors.

Perceived behavioral control is based on how difficult one perceives a task to be in the context of available opportunities and resources (Randall, 1994). Therefore, Chang (1998) emphasized that authorities have a responsibility to minimize the opportunities to engage in unethical behaviors in order to reduce individuals' perception of their behavioral control and diminish the intentions of such behaviors. Similarly, athletes' perceptions of the difficulty of engaging in deviant behaviors influence the intentions that support those actions. Thus, it is advisable for authorities in sports settings, such as coaches, to restrict the availability of opportunities for athletes to perform unethical acts.

Moral obligation, defined as a sense of responsibility to behave morally, is included in the ethical decision-making process, and the perceived importance of an ethical issue influences moral obligation in this process (Beck and Ajzen, 1991; Leonard et al., 2004; Haines et al., 2008). Therefore, to appeal to athletes' moral obligation concerning unethical behaviors, it is suggested that they be disciplined and educated to increase their perception of the importance of morality in deviant behaviors. Providing athletes with the tools to identify ethical issues in sports settings and the magnitude of the influence these issues will have in their lives will be critical and beneficial components of the education process.

Interestingly, it was found that the intentions of athletes did not predict deviant behaviors before grouping based on impulsivity level. Although intention is the most important factor in TPB to predict one's behavior, even Ajzen said that there is an intention–behavior discrepancy that does not lead to action triggers even if intention is high (Ajzen et al., 2004). Moreover, a lot of study dealing with intention–behavior gap (Hagger and Chatzisarantis, 2008; Rhodes and de Bruijn, 2013) described it as the result of the gap between the pre-action intention and post-action determination. Since we investigated deviant behaviors 3 months after measuring the intentions, intention-behavior inconsistency may have occurred. Moreover, deviant behaviors in sports could be impulsive factors in the decision-making process. Indeed, the existing study confirmed that impulsivity moderates the impact of intention on actual behaviors (Churchill and Jessop, 2010), and after dividing groups based on impulsivity level in our study, we found that intention significantly predicts deviant behavior.

Factors influencing deviant behaviors can be divided into personal and situational categories. Personal factors include personality or psychological factors, whereas situational factors pertain to an individual's environment, such as the family, school, and social environments. This study was based on the belief that impulsivity plays a role in transforming the intention to engage in deviant behaviors into actual deviant acts. Our results revealed that high impulsivity increases the likelihood that an intention for deviant behavior will materialize into an actual act. Research on dishonesty has shown that highly impulsive individuals are restless, thrill-seeking, tend to immediately act on their thoughts, and lack planning abilities in solving problems (Patterson, 1986). As such, systematically stabilizing teenagers' impulsivity is critical for reducing deviant behaviors in youths, which is consistent with the theme of the present study which examines the association between impulsivity and deviant behaviors in sports (Booth and Zhang, 1996). These findings suggest that techniques for reducing impulsivity and increasing self-regulation need to be taught to young people early in their education in order to reduce deviant behaviors in sports among young athletes (Gailliot and Baumeister, 2007; Friese and Hofmann, 2009). According to the existing work (Toering et al., 2011), planning before a certain behavior, consistent self-monitoring, and evaluating the behavioral consequences of potential actions are helpful tools one can employ to increase self-regulation ability. Thus, developing self-regulation among the athletes by providing them with practical instruction in processes such as planning, self-monitoring, and evaluating consequences could diminish their impulsive, unethical behaviors in sports.

Furthermore, in systematic approaches to behavior, “the behavior change wheel” (Michie et al., 2011) was introduced to understand, characterize, and design interventions for behavioral change. It provides theory-based concepts using capability, opportunity, and motivation to generate behaviors and in turn be influenced by behaviors (the COM-B system). Using this system we can reduce deviant behavior along with the intervention of education (providing information to demote deviant behavior), persuasion (using imagery about opponents who suffered to motivate a decrease in deviant behavior), and incentivization (using prize to induce attempts to stop deviant behavior). It is believed that these interventions can positively affect the athletes' attitude, subjective norms, perceived behavior control, and moral obligation toward deviant behavior. Further research is required to verify that the behavior change wheel can be applied to reduce deviant behavior in sports.

In addition, the independent sampled *t*-tests by groups (genders, school levels, 1:1 contact sports vs. non-contact sports), involving moral obligation and deviant behavior were conducted with the data in the present study. The results showed that the female students had higher moral obligation and lower deviant behavior compared to the male students. Middle school students had higher moral obligation than high school students, and deviant behaviors among middle school students were lower than among high school students. Contact sports exhibited lower moral obligation and higher actual deviant behaviors when compared to non-contact sports. All previous results were statistically significant (*p* < 0.005). However, there were no significant differences between groups in SEM analysis. Although this analysis is not included in the Results section, it provides information for follow-up studies. Further studies will be needed to examine these results in more detail.

This study is important because it is the first study on deviant behaviors in sports grounded in an established theory that incorporates an additional variable. Covariance structure analyses are often used to address the problem of equivalent models (MacCallum et al., 1993), where models have identical fit but different paths. The problem of equivalent models is addressed here because the models are grounded in established theory.

## Limitations

The present study focused on middle and high school individual-sport athletes, so the findings cannot be generalized for team-sport athletes. Ethical decision-making among team-sport athletes is usually influenced by the teams' ethical standards, and therefore may differ from ethical decision-making among individual-sport athletes (Shields and Bredemeier, 2001); therefore, deviant behaviors in team sports must be examined in future research. Additionally, the role of self-control in reducing the facilitative effect of impulsivity (Friese and Hofmann, 2009) on deviant behaviors will require further study in order to improve and complete our understanding of these behaviors.

The research model employed in the present study may have incorporated social desirability-based responses or bias because only the athletes' perceptions about their own deviant behaviors were measured. Future research should consider the perceptions of unethical behaviors of teammates or coaches in sports to provide a multi-dimensional perspective.

## Conclusion

The present study examined the association between young athletes' impulsivity and deviant behaviors in sports based on the extended TPB at two time points. The results indicated that the perceived behavior control and an individual's sense of moral obligation greatly influence the intention of engaging in deviant behaviors. Further, the study suggested that limiting impulsivity is key to reducing deviant behaviors in youths. To reduce deviant behaviors in sports among middle and high school athletes, coaches or other significant authority figures should focus on cultivating a sense of moral obligation and a spirit of fair play, rather than encouraging a win-at-all cost philosophy. In addition, providing athletes with tools to improve their self-regulation abilities will enable them to control their impulsivity, which in turn should help prevent engagement in deviant behaviors in sports. These efforts will help promote moral growth in young athletes, and contribute to a more congenial and ethical culture in sports settings.

## Data Availability Statement

The original contributions presented in the study are included in the article/supplementary material, further inquiries can be directed to the corresponding author/s.

## Ethics Statement

The studies involving human participants were reviewed and approved by Seoul National University IRB (No. 2103/001-007). The patients/participants provided their written informed consent to participate in this study.

## Author Contributions

SK, IK, and KL: conceptualization. SK: methodology, formal analysis, and writing—original draft preparation. IK: writing—review and editing. KL: supervision. All authors have read and agreed to the published version of the manuscript.

## Conflict of Interest

The authors declare that the research was conducted in the absence of any commercial or financial relationships that could be construed as a potential conflict of interest.

## Publisher's Note

All claims expressed in this article are solely those of the authors and do not necessarily represent those of their affiliated organizations, or those of the publisher, the editors and the reviewers. Any product that may be evaluated in this article, or claim that may be made by its manufacturer, is not guaranteed or endorsed by the publisher.
